# Introduction of Japanese Encephalitis Virus Genotype I, India

**DOI:** 10.3201/eid1702.100815

**Published:** 2011-02

**Authors:** Pradip V. Fulmali, Gajanan N. Sapkal, Sulabha Athawale, Milind M. Gore, Akhilesh C. Mishra, Vijay P. Bondre

**Affiliations:** Author affiliations: National Institute of Virology, Pune, India (P. V. Fulmali, G.N. Sapkal, S. Athawale, A.C. Mishra, V.P. Bondre);; National Institute of Virology, Gorakhpur, India (M.M. Gore)

**Keywords:** Japanese encephalitis virus, vector-borne infections, genotype I, acute encephalitis syndrome, sequencing, viruses, letter

**To the Editor:** Seasonal outbreaks of fatal acute encephalitis syndrome (AES) occur regularly in several parts of India. Japanese encephalitis virus (JEV) has been the major and consistent cause of these outbreaks in the Gorakhpur region of Uttar Pradesh State, accounting for ≈10%–15% of total AES cases annually (*1**–**3*). In India, vaccinations against Japanese encephalitis (JE) are administered in areas where the disease is hyperendemic, including Gorakhpur, and AES cases are regularly investigated to clarify the effects of vaccination. Currently, >2,000 patients with AES are admitted each year to Baba Raghav Das Medical College, Gorakhpur.

JEV is classified into 5 genotypes. Genotype III (GIII) is widely distributed in Asian countries, including Japan, South Korea, the People’s Republic of China, Taiwan, Vietnam, the Philippines, India, Nepal, and Sri Lanka (*4*). However, during the past decade, JEV GI has been introduced into South Korea, Thailand, and China and has replaced the GIII strains that had been circulating in Japan and Vietnam during the mid-1990s (*5*). Until 2007, all known JEV strains isolated in India belonged to GIII (*2**–**4**,**6*).

The JE-endemic Gorakhpur region recorded a sudden increase in AES cases during September–November 2009. Clinical specimens collected from 694 hospitalized patients were examined for JEV infection by JEV-specific immunoglobulin M capture ELISA (*7*). Clinical specimens comprising 115 (16.6%) cerebrospinal fluid (CSF) specimens and 114 (16.4%) serum specimens showed recent JE infection among 158 (22.7%) of the case-patients.

All CSF specimens were processed for JEV genome detection by diagnostic reverse transcription–PCR (RT-PCR), which amplified the nucleocapsid-premembrane genes (*7*). Additionally, envelope (E) gene–specific primers designed from Indian JEV isolate GP78 (GenBank accession no. AF075723) were used for E gene amplification.

The diagnostic RT-PCR detected JEV in 66 (9.5%) of 694 CSF specimens (GenBank accession nos. HM156543–HM156569, HM156573–HM156611). Among them, 27 sequences differed from the remaining 39, with only 86.2%–88.7% nt identity. The group of 27 sequences showed 99.2%–100% nt identity with each other and a high of 95.0% nt identity with Japanese GI swine JEV isolate (AB241119). The 39 sequences showed 94.2%–100.0% nt identity with each other and with other Indian GIII JEV strains. These findings indicate that both GI and GIII JEV strains circulate in the Gorakhpur region.

The E gene sequence was amplified from 4/66 JEV-positive CSF samples (GenBank accession nos. HM156570–HM156572, HM156612). A comparison of E gene nucleotide sequences those of other JEV isolates from the region showed that 1 E gene sequence belonged to GIII and the other 3 to GI (Figure). The 3 GI E gene sequences were most similar (98.6%) with Japanese isolate 95–167/1995/swine (AY377579), followed by 98.5% similarity with Korean isolate K96A07/1996 (FJ938219). The single GIII E gene sequence showed 95.6%–99.8% nt identity with other Indian GIII isolates with the highest similarity (99.8% nt identity) with the 014178 (EF623987) JEV isolate from the 2001 Uttar Pradesh outbreak. Analysis of the E gene sequence amplified from 2 JEV isolates, obtained by injecting 29 CSF samples, positive by RT-PCR, into baby hamster kidney cells, showed 100.0% nt identity with sequences directly amplified from respective CSF specimens (*3*). Phylogenetic analysis of these E gene sequences, along with other 55 GenBank sequences, confirmed that 3 sequences belonged to JEV GI, and 1 belonged to GIII (Figure).

**Figure:**
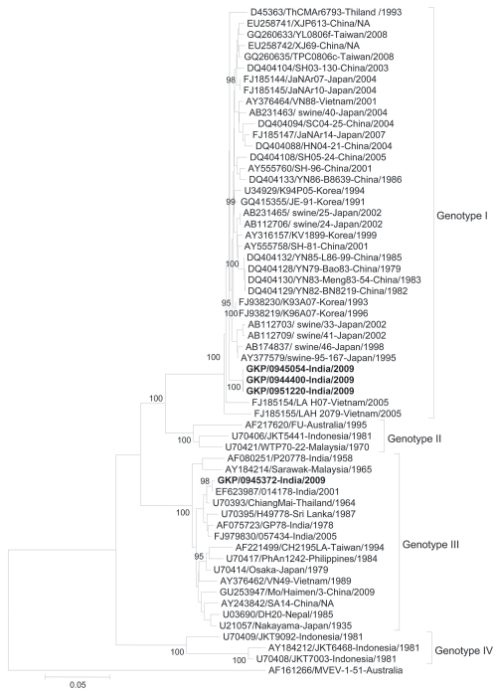
Phylogenetic tree constructed by using a 1,381-nt Japanese encephalitis virus (JEV) envelope sequence directly amplified from cerebrospinal fluid specimens collected during the acute phase of illness from hospitalized acute encephalitis syndrome patients, India, September–November 2009. Multiple sequence alignment and phylogenetic analysis were conducted by using ClustalW software (www.ebi.ac.uk/Tools/clustalw2/index.html) and MEGA version 4 (www.megasoftware.net). The phylogenetic tree was constructed by the neighbor-joining method and the maximum composite likelihood model. The robustness of branching patterns was tested by 1,000 bootstrap pseudo replications. Sequences obtained in this study are indicated in **boldface**. Genotypes are indicated on the right. Viruses were identified by using the nomenclature of accession number–strain name–country of origin/year of isolation. Bootstrap values are indicated above the major branch. The tree was rooted within the Japanese encephalitis serogoup by using Murray Valley encephalitis virus (AF161266) and 55 JEV sequences from GenBank were used in the analysis. Scale bar indicates nucleotide substitutions per site.

The first JE outbreak in the Gorakhpur region was documented during 1978. Since then, JE epidemics have occurred regularly (*8*). This study demonstrated simultaneous detection and isolation of GI and GIII JEV strains from AES case-patients. Documented clinical symptoms among patients infected with the 2 strains were indistinguishable.

GI JEV isolates from India share close genetic relationship with GI strains from Japan and Korea. In India, JEV neutralizing antibodies have been detected in 179 (34.8%) of 514 birds, including pond herons and cattle egrets, indicating a possible role in virus maintenance (*9*). Large perennial lakes, swamps, and rice fields provide a wintering and staging ground for several migratory waterfowl; such areas also favor breeding and survival of mosquitoes (*10*). Considering these conditions, GI JEV may have been introduced into India through migratory birds, as it has in other Asian countries (*5*). However, the exact mode of introduction of GI JEV into India is not known, and further studies are needed to determine the role of migratory birds in JE transmission.

This study suggests the recent introduction of JEV GI strain in India. Simultaneous detection of GI and GIII strains indicates their co-circulation and association with human infections in Gorakhpur region. Because the live attenuated JE vaccine used in India is derived from GIII strain SA14–14–2, the efficacy of the vaccine to protect against GI JEV must be carefully evaluated. Thus, the genetic and antigenic variation among JEV strains circulating in India should be monitored to determine effects on JE epidemiology and ongoing vaccination efforts. Additionally, the expansion of GI JEV into other parts of India should be continuously tracked.
